# Structural architecture of collagen and collagen-fibronectin networks is associated with the invasive behavior of liver cancer cells

**DOI:** 10.1007/s13402-026-01235-0

**Published:** 2026-06-12

**Authors:** Alexander Hayn, Madlen Matz-Soja, Thomas Berg, Florian van Bömmel

**Affiliations:** Division of Hepatology, Department of Medicine II, Leipzig University Medical Center, Liebigstr. 19, 04103 Leipzig, Germany

**Keywords:** Extracellular matrix, Fibrosis, Hepatocellular carcinoma, Intrahepatic cholangiocarcinoma

## Abstract

**Purpose:**

The remodeling of the extracellular matrix (ECM) is a key feature of tumor development in the liver, particularly in hepatocellular carcinoma (HCC) and intrahepatic cholangiocarcinoma (iCCA). Fibrosis and cirrhosis are risk factors for tumorigenesis and define serious structural changes in the ECM. Fibrotic-induced collagen and fibronectin alter the stiffness and heterogeneity of the ECM. A stiffened and heterogeneous ECM promotes the proliferation, migration, and invasion of tumor cells. Direct effects of structural changes in the ECM on HCC/iCCA cancer cells are insufficiently studied.

**Methods:**

HCC and iCCA cells were examined in contact with differently structured collagen and collagen-fibronectin networks. The structural architecture of the networks was defined by combining the normalized stiffness, pore size, and network distribution values as the ECM Architectural Index (EAI). The effects of cell-matrix interactions on cell stiffness, invasiveness, and the ability to interact with network structures through fiber displacement in relation to network architecture were determined.

**Results:**

Increased EAI caused a decrease in cell stiffness, an increase in cell invasiveness, and altered fiber displacements. Different effects on HCC and iCCA cells depending on the EAI were identified. High EAI resulted in low cell stiffness and high cell invasion across cancer cell types.

**Conclusions:**

We present a model system that is applicable to identify structurally induced influences of the extracellular matrix on cancer cells and to investigate the risk factor of structural changes in the tumor environment.

**Supplementary information:**

The online version contains supplementary material available at 10.1007/s13402-026-01235-0.

## Introduction

Primary liver cancers as hepatocellular carcinoma (HCC) and intrahepatic cholangiocarcinoma (iCCA) are malignancies with poor prognosis and rising global incidence [1–3]. The prognosis of these cancers is strongly determined by liver tissue invasion of tumor cells and development of metastasis [2–4].

The liver tissue’s framework for its structural and functional integrity constitutes of the matrisome, a complex and dynamic extracellular matrix (ECM). The ECM is composed of over 300 macromolecules that can be grouped into polyglycans, glycoproteins and collagens [5, 6]. In healthy tissues, the dynamic ECM supports physiological functions and limits tumorigenesis [7]. During liver disease progression, chronic injury triggers a wound-healing response. In this process, activated hepatic stellate cells undergo transdifferentiation into myofibroblasts that increase secretion of collagen and fibronectin, leading to remodeling processes of the ECM [8–10]. As a consequence, fibronectin and basement membrane collagens such as type I, III, IV, and VI are elevated to varying degrees, with collagen type I being the most predominant type associated with progression of cirrhosis.

ECM remodeling can contribute significantly to both, tumor initiation and progression by different mechanisms [4, 5, 11–18]. Thus, tumor development can be triggered by mechanical stress, combined with inflammation-induced fibrosis and fibrosis-induced inflammation, which interact in a chronic inflammatory-fibrotic loop, that promotes genomic instability and impairs DNA repair [12, 19, 20]. Mechanical stress translates to biochemical signal through several pathways, including integrin-mediated mechanotransduction, which increases the cell’s internal contractility via Rho/ROCK signaling, the Hippo pathway via the transcriptional co-activators YAP and TAZ, fibrosis-driven inflammation, or pro-growth cytokines like TGF-β and HGF that promote mitogenic signaling [21–24]. In HCC, remodeled, interconnected fibrous ECM components form a fibrotic capsule that disrupts vascularization, leads to the loss of normal sinusoids, and increases structural heterogeneity [25]. Fibronectin, a glycoprotein in the ECM of hepatic tissue that accumulates strongly during chronic injuries is likewise associated with HCC development [16–18]. Once a tumor is established, increased ECM density and alignment can facilitate tumor progression. Especially the disorganization of the ECM as a functional interplay of structural, mechanical, biochemical, and feedback-mediated dynamic disorganization results in an increased cancer cell sensitivity to mechanical stress, and rigid collagen fibers can act as physical tracks for cancer cell migration [12, 13]. Although those mechanisms are believed to foster tumor development and progression, the relationship between cirrhosis, matrisome abnormalities, and liver cancer is complex and remains incompletely understood.

Measuring liver stiffness has evolved into a standard clinical procedure for staging liver disease and establishing patient prognosis [26]. Moreover, increased liver stiffness is associated with a higher risk of developing primary liver carcinomas and a higher rate of metastasis/invasion of liver tumors [27]. Collagens, as central components of the ECM, are increasingly viewed as high-value targets for experimental therapies. [28, 29]. Given the tight link between mechanical rigidity, structural remodeling, and the presence of collagen and fibronectin in the progression of liver cancer [30, 31], our study investigates how the heterogeneity of collagen- and fibronectin-based ECM structures affects cell-matrix interactions. Specifically, we explore how these mechanical variations influence tumor cell invasion and drive significant alterations in individual cell stiffness.

**Figure:**
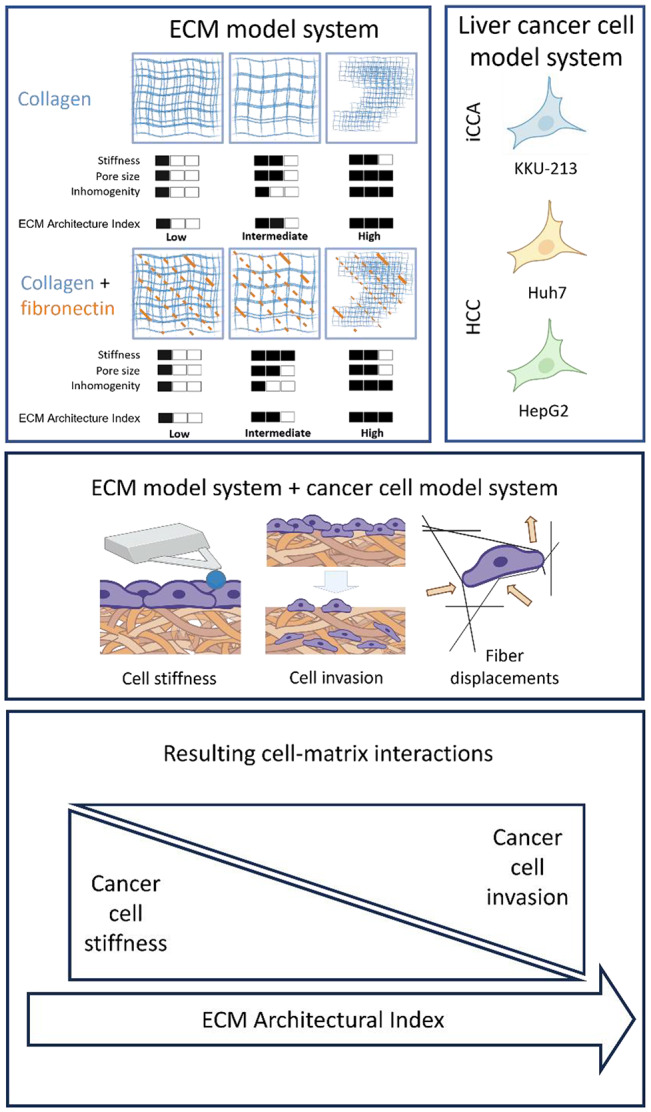
Collagen type I networks with and without fibronectin were produced. The properties of these networks were examined biophysically. The normalized stiffness, pore size, and network distribution values were combined to form an ECM Architectural Index (EAI). A liver cancer cell model system for HCC and iCCA consisting of KKU-213, Huh7, and HepG2 cells was brought into contact with the various networks. The cell-matrix interactions were examined in terms of cell stiffness, cancer cell invasiveness, and fiber displacements. Intermediate and high EAI reduces cell stiffness, increases cancer cell invasiveness, and thus contributes to increased cancer cell aggressiveness. Created with BioRender.com

## Results

### Biophysical differentiation of the ECM model systems

#### Differences in the collagen network structures

**Fig. 1:**
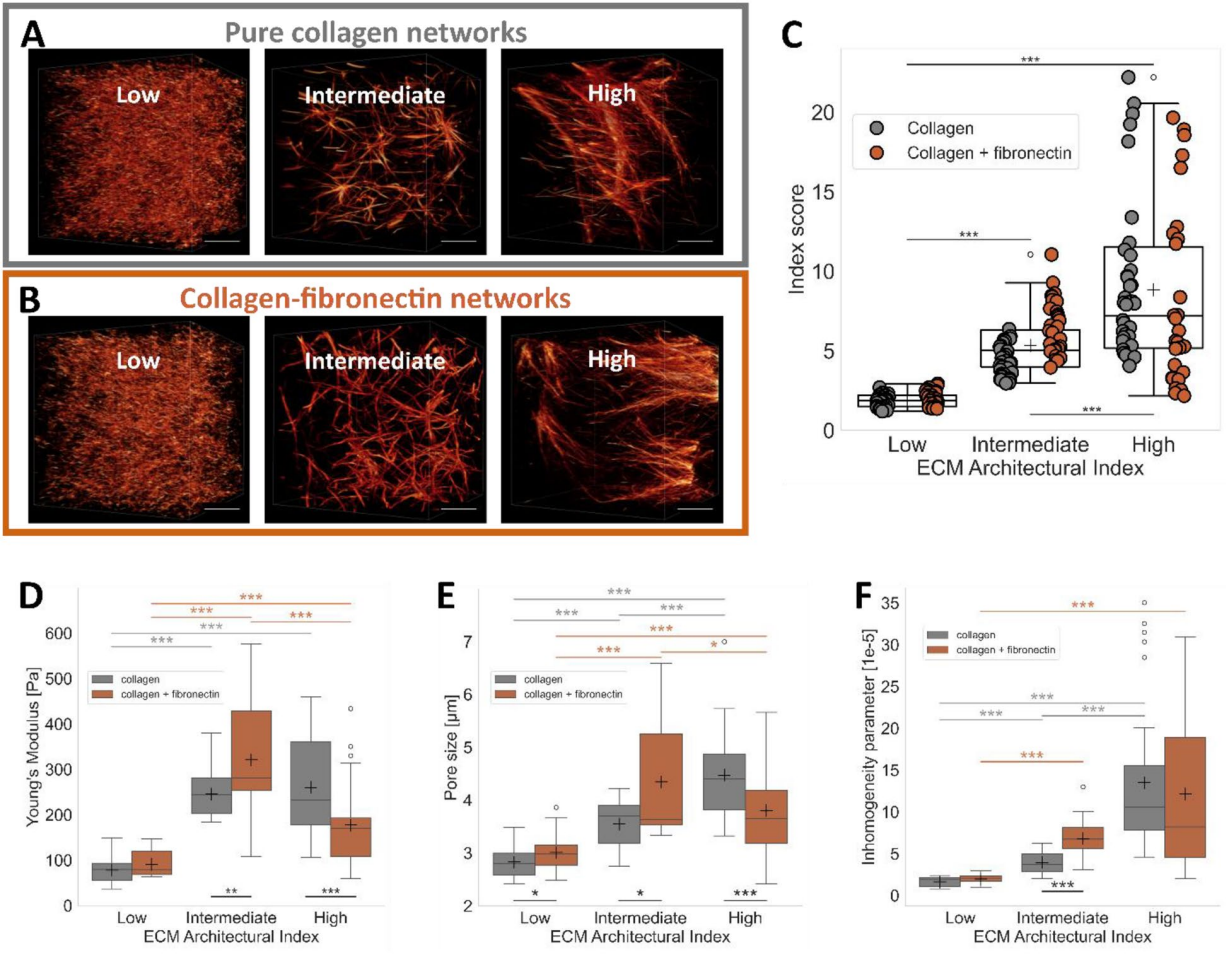
Network structure of ECM model systems. (**A**) Visualization of fluorescence-stained collagen and (**B**) collagen-fibronectin z-stacks (dimensions are 100 µm in x, y and z) taken with a confocal laser scanning microscope, scale bars are 20 µm. ‘low’ collagen networks and ‘low’ collagen networks with fibronectin consist of relatively dense structures made by short collagen fibrils. ‘intermediate’ networks from pure collagen and with fibronectin addition constituted by long fibrils and node-like structures. ‘high’ collagen networks and ‘high’ collagen-fibronectin networks consists of fiber assemblies forming huge node-like structures. (**C**) Parameterized EAI as a classification of the diversity of the networks based on the merging of the n-fold changes of stiffness, pore size and inhomogeneity of the networks. (*n* = 31 for ‘low’ collagen, *n* = 29 for ‘low’ collagen + fibronectin, *n* = 34 for ‘intermediate’ collagen, *n* = 29 for ‘intermediate’ collagen + fibronectin, *n* = 34 for ‘high’ collagen, *n* = 29 for ‘high’ collagen + fibronectin. (**D-F**) Biophysical and intrinsic parameters networks constituted from pure collagen (grey boxes) and collagen with additional fibronectin (red boxes) (**D**) Stiffness of ‘low’ (n_SC_ = 38, n_SFN_ = 32), ‘intermediate’ (n_SC_ = 41, n_SFN_ = 39) and ‘high’ (n_SC_ = 58, n_SFN_ = 40) networks with and without fibronectin. (**E**) Pore size and (**F**) inhomogeneity describing intrinsic characteristics of the ‘low’ (n_PIC_ = 31, n_PIFN_ = 29), ‘intermediate’ (n_PIC_ = 34, n_PIFN_ = 29) and ‘high’ (n_PIC_ = 34, n_PIFN_ = 29) collagen and collagen-fibronectin networks. Significance notions (derived from Mann-Whitney U test) express ***p ≤ 0.001, **p ≤ 0.01, *p ≤ 0.05. Kruskal–Wallis test revealed *** significance for all conditions. Boxes are confined by 25th and 75th percentile, horizontal lines are the medians, whiskers describe 5th and 95th percentile. The mean value is indicated by a + symbol. Number of replicated measurements are indicated as n_SC_ (stiffness - pure collagen), n_SFN_ (stiffness - collagen + fibronectin), n_PIC_ (pore size and inhomogeneity - pure collagen), n_PIFN_ (pore size and inhomogeneity - collagen + fibronectin). Data contain at least 5 independent experiments

Collagen model systems termed ‘low’ visually appeared very uniform in terms of the distribution of small fibrillary structures (Fig. 1A and Supplementary video SV1). The addition of fibronectin to the ‘low’ collagen systems prior polymerization did not alter this (Fig. 1B and Supplementary video SV2). Overall, the fibronectin containing ‘low’ networks appeared slightly looser, compared to the pure collagens in the ‘low’ ECM model system. So called ‘intermediate’ networks visually appeared to be constituted by long thin fibrils and large nodal structures (Fig. 1A and Supplementary video SV3). Fibronectin addition to ‘intermediate’ networks seemed to alter the fibrillary structures to thicker fibrils and less finely structured nodular constructs compared to the ‘intermediate’ networks without fibronectin (Fig. 1B and Supplementary video SV4). Fibronectin can cross-link with collagen fibers [32] and affects the structure in the form of fiber alignment and increases pore size [33]. Networks classified as ‘high’ exhibit more irregular structural distribution compared to ‘low’ and ‘intermediate’ networks with and without fibronectin (Fig. 1A and Supplementary video SV5). Short, fine fibrillar structures and much thicker fiber-like collagen constructs together formed strongly pronounced node-like structures. ‘high’ collagen networks with fibronectin also appeared to have distinctly more inhomogeneously distributed structures than in ‘low’ and ‘intermediate’ networks with and without fibronectin (Fig. 1B and Supplementary video SV6). The node-like structures present in the networks appeared slightly less massive than in ‘high’ collagens without added fibronectin. The node-like structures in ‘high’ networks with and without fibronectin had prominent structures that are aligned in themselves and oriented towards the node-like structures.

#### Definition and categorization of the ECM model systems

The ECM Architectural Index (EAI) was defined in order to distinguish networks based on their properties that affect network structure (Fig. 1C). The EAI is derived from the Euclidean norm of normalized stiffness, pore size, and values of the spatial distribution of network structures (termed inhomogeneity) and thus enabled the description of ECM architecture and the classification into ‘low, ‘intermediate’ and ‘high’. (Table 1).

**Table 1:** Structural characteristics of different ECM model systems. Values containing data for the boxes (Young’s Modulus (Fig. 1D), pore size (Fig. 1E) and inhomogeneity (Fig. 1F)) in form of 25 percentile (25%), median (50%) and the 75^th^ percentile (75%). N is the number of independent repetitions; n is the number of the experimental procedures

EAI	ECM-protein	Young’s Modulus [Pa]					Pore size [µm]					Inhomogeneity [1e-5]					Index Score			
		25%	50%	75%	N	n	25%	50%	75%	N	n	25%	50%	75%	N	n	25%	50%	75%	n
Low	Collagen	56	80	93	5	38	2,6	2,8	3,0	9	31	1,1	1,9	2,1	8	31	1,5	1,9	2,1	31
	Collagen + fibronectin	68	80	120	5	32	2,8	3,0	3,2	9	29	1,7	2,1	2,3	8	29	1,7	2,0	2,4	29
Intermediate	Collagen	202	245	281	5	41	3,2	3,7	3,9	8	34	2,8	3,7	5,0	6	34	3,4	4,1	5,1	34
	Collagen + fibronectin	253	282	428	5	39	3,5	3,6	5,2	6	29	5,6	6,7	8,1	6	29	5,1	6,5	7,7	29
High	Collagen	178	232	361	6	58	3,8	4,4	4,9	8	34	7,8	10,6	15,5	7	34	6,0	8,0	10,8	34
	Collagen + fibronectin	108	170	193	5	40	3,2	3,6	4,2	8	32	4,6	8,2	18,9	7	29	3,6	5,6	12,0	29

The stiffness of ‘intermediate’ and ‘high’ networks was significantly higher compared to ‘low’ networks for pure collagen networks as well as for collagen-fibronectin networks. The addition of fibronectin prior to polymerization of the networks produced networks with significantly higher stiffness compared to the pure collagen networks. However, the stiffness of the ‘high’ network with fibronectin was significantly lower compared to their pure collagen network counterparts. The stiffness of ‘low’ networks is independent of fibronectin addition with a slightly trend towards higher stiffness for collagen-fibronectin networks (Fig. 1D).

Pore size determination revealed significant differences between networks made of pure collagen and collagen-fibronectin networks for all determined ECM model systems. The pore size of pure collagen networks was significantly larger, ranging from ‘low’ to ‘intermediate’ to ‘high’ networks. The pore size of collagen-fibronectin networks was significantly larger ranging from ‘low’ to ‘intermediate’ networks and significantly smaller from ‘intermediate’ to ‘high’ networks (Fig. 1E).

The inhomogeneity of pure collagen networks significantly increased from ‘low’ networks to ‘intermediate’ networks and further increased significantly to ‘high’ networks. For collagen-fibronectin networks the inhomogeneity of ‘intermediate’ and ‘high’ networks was significantly higher than the inhomogeneity for ‘low’ networks. In the case of ‘intermediate’ networks, the observed inhomogeneity of collagen-fibronectin networks is significantly higher than in pure collagen networks. (Fig. 1F).

Overall, the ‘low’ ECM model systems with and without fibronectin represent a suitable control condition for investigating the effects of structural changes on cell-matrix interactions due to their less pronounced and weakly influenced structural characteristics (in the form of low stiffness, small pore size, and low inhomogeneity).

### Cell stiffness determination due to interaction with ECM model systems

**Fig. 2:**
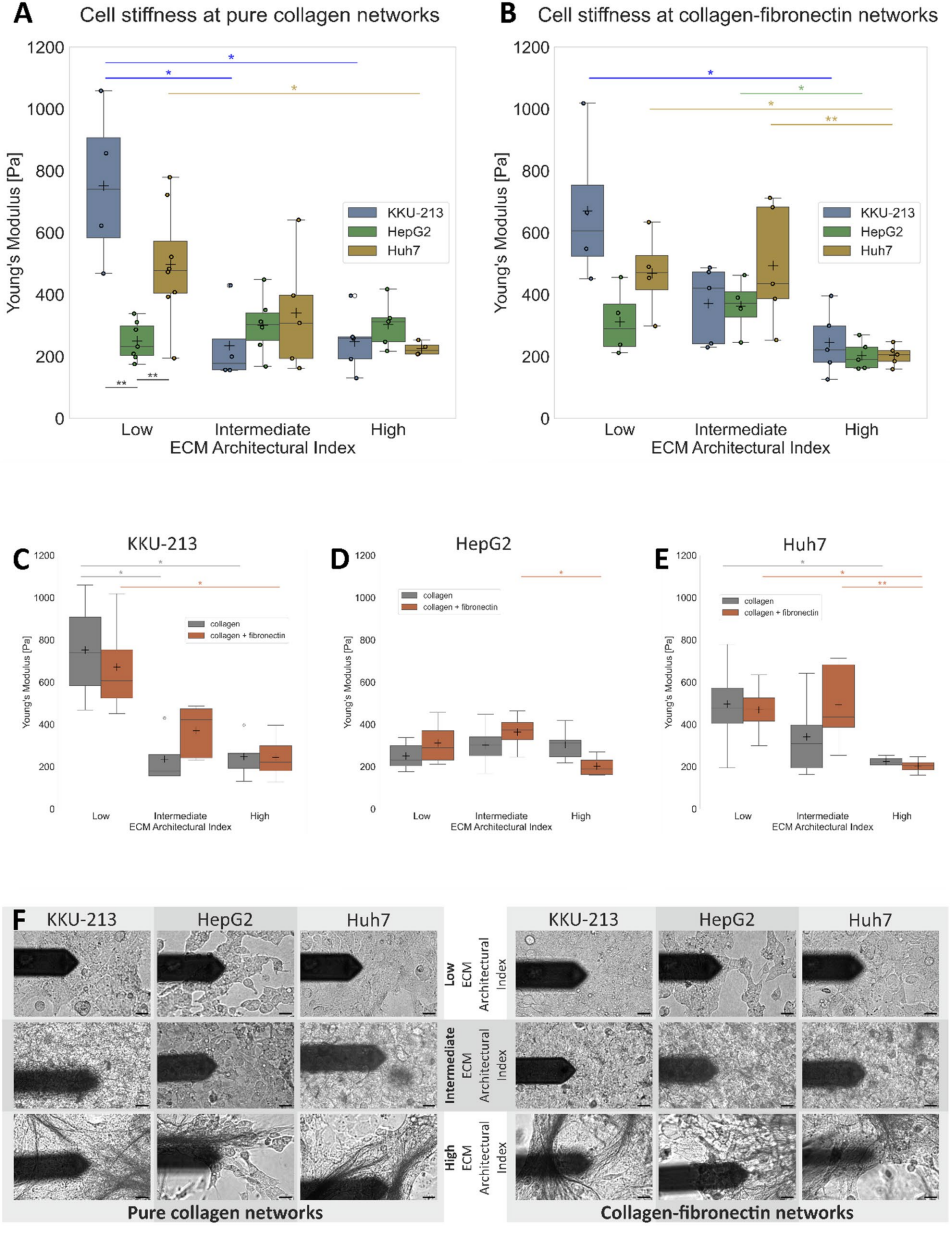
Structural changes of the ECM alter cellular stiffness. (**A**) Cell stiffness for cells adhered at pure collagen matrices with ‘low’ (n_KKU_ = 13, N_KKU_ = 4, n_Huh_ = 24, N_Huh_ = 8, n_Hep_ = 21, N_Hep_ = 7), ‘intermediate’ (n_KKU_ = 13, N_KKU_ = 4, n_Huh_ = 13, N_Huh_ = 5, n_Hep_ = 22, N_Hep_ = 6) and ‘high’ (n_KKU_ = 15, N_KKU_ = 5, n_Huh_ = 15, N_Huh_ = 4, n_Hep_ = 20, N_Hep_ = 5) EAI. (**B**) Cell stiffness for cells adhered at collagen matrices with additional fibronectin with ‘low’ (n_KKU_ = 15, N_KKU_ = 4, n_Huh_ = 11, N_Huh_ = 4, n_Hep_ = 19, N_Hep_ = 4), ‘intermediate’ (n_KKU_ = 15, N_KKU_ = 5, n_Huh_ = 15, N_Huh_ = 5, n_Hep_ = 21, N_Hep_ = 4) and ‘high’ (n_KKU_ = 13, N_KKU_ = 5, n_Huh_ = 19, N_Huh_ = 5, n_Hep_ = 24, N_Hep_ = 5) EAI. Data of the biological replicates N presented as combined box plots and strip plots. (**C-E**) Cell-specific differences in cell stiffness for cells in contact with various collagen networks with and without fibronectin content (**C**) presented for KKU-213 cells, (**D**) HepG2 cells and (**E**) Huh7 cells. Figure (**F**) shows representative images in a panel for adherent KKU-213 (left columns), HepG2 (middle columns), and Huh7 (right columns) cells on pure collagen networks (left matrix) and collagen-fibronectin networks (right matrix) and the different networks in the form of ‘low’ (top row), ‘intermediate’ (middle row), and ‘high’ (bottom row) EAI. The cantilever (black object) used for cell stiffness measurements with the AFM is visible. The scale bars correspond to 50 µm. Boxes are confined by 25th and 75th percentile, horizontal lines are the medians, whiskers describe 5th and 95th percentile. The mean value is indicated by a + symbol. Significance notions (derived from Mann-Whitney U test) express **p ≤ 0.01, *p ≤ 0.05. Kruskal–Wallis test revealed ** significance for pure collagen as well as for collagen fibronectin networks. Number of replicated measurements (technical replicates with 5 × 5 indentations each) are indicated as n_KKU_ (KKU-213 cells), n_Huh_ (Huh7 cells), n_Hep_ (HepG2 cells). Number of independent repetitions (biological replicates) are indicated as N_KKU_ (KKU-213 cells), N_Huh_ (Huh7 cells), N_Hep_ (HepG2 cells)

The cell stiffness measured with an atomic force microscope (AFM) and expressed by Young’s modulus for KKU-213 cells in contact with pure collagen networks was significantly higher at ‘low’ networks compared to the stiffness at the respective networks with ‘intermediate’ and ‘high’ EAI. The stiffness of Huh7 cells was significantly higher in contact with ‘low’ compared to the cell stiffness in contact with ‘high’ pure collagen networks. Within the respective networks based on pure collagen, significant differences in stiffness were measured between the observed cell lines in the ‘low’ system. Huh7 and KKU-213 cells had significantly higher cell stiffness than HepG2 cells. (Fig. 2A).

In the case of fibronectin-containing collagen matrices, the cell stiffness of KKU-213 cells in contact with ‘low’ networks was higher than in contact with ‘intermediate’ networks and significantly higher than in contact with ‘high’ networks. The stiffness of Huh7 cells was significantly lower in contact with ‘high’ networks compared to less and ‘intermediate’ collagen-fibronectin networks. HepG2 cells differ significantly in terms of a higher Young’s Modulus in contact with ‘intermediate’ networks and a lower Young’s Modulus in contact with ‘high’ networks of collagen and fibronectin compared to the cell stiffness in contact with ‘low’ networks (Fig. 2B).

The expression of cell stiffness in comparison between collagen and collagen-fibronectin networks revealed a higher cell stiffness for all investigated cell lines under the influence of matrix-bound fibronectin in ‘intermediate’ ECM model systems. In ECM model systems with ‘high’ EAI this effect was tendentially reversed (Fig. 2C–E).

Overall, the cell stiffness of KKU-213 and Huh7 cells was significantly higher in contact with ‘low’ compared to ‘high’ networks, while the stiffness of HepG2 cells is less strongly affected by the network EAI. Finally, in networks with ‘high’ EAI with and without fibronectin, the cell stiffness of all cell lines examined was at a similar (low) level (Table 2).

### Liver cancer cell invasion in different ECM model systems

**Fig. 3:**
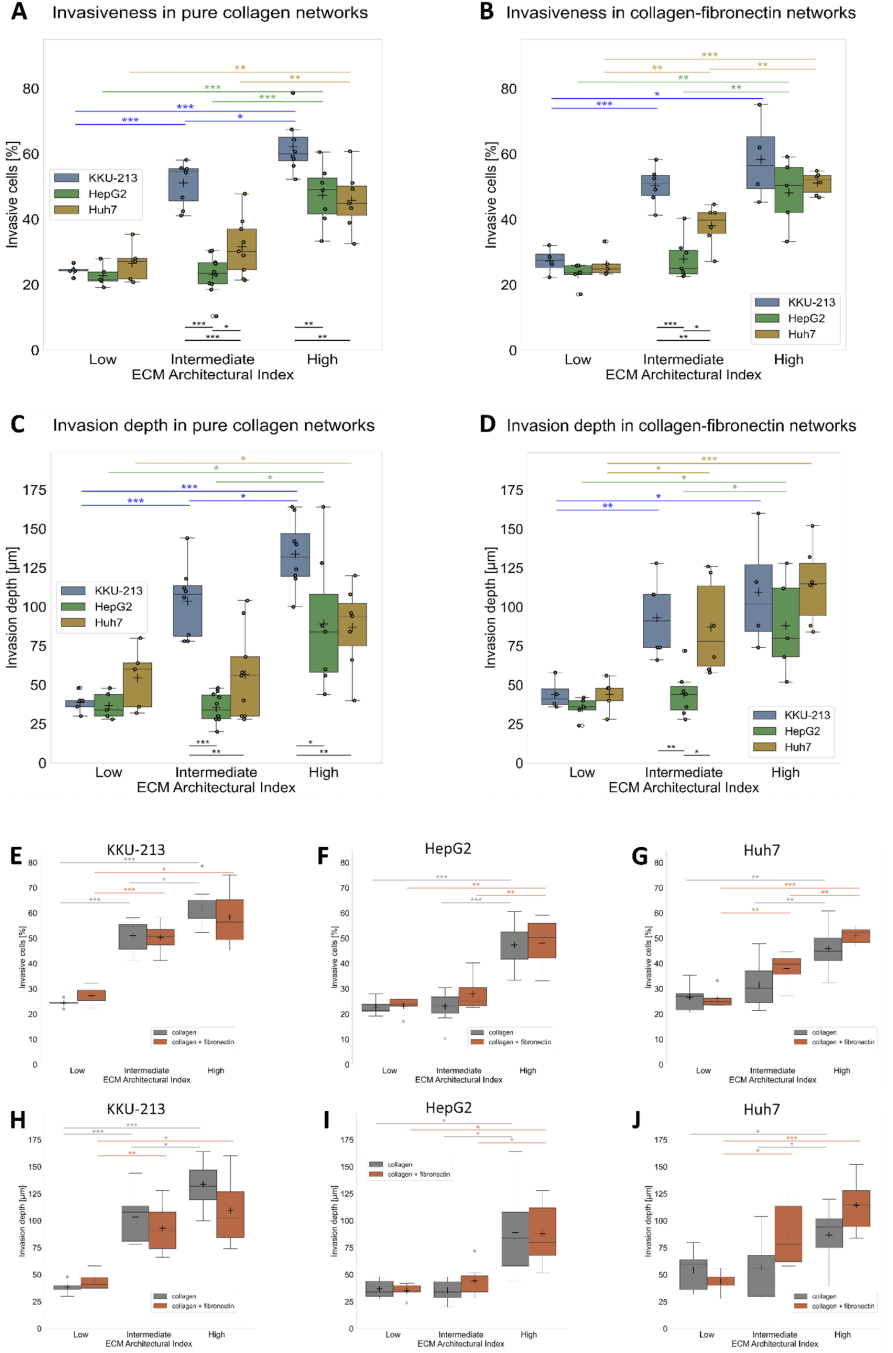
ECM structure impacts liver cancer cell invasion. (**A-B**) The ratio of invaded cells and non-invasive cells is defined as invasiveness. (**C-D**) The depth to which cancer cells were found in the respective networks after 72 hours is termed invasion depth. (**A**) Invasiveness in pure collagen networks of different liver cancer cells in ‘low’ (N_KKU_ = 5, N_Huh_ = 5, N_Hep_ = 5),’ intermediate’ (N_KKU_ = 8, N_Huh_ = 9, N_Hep_ = 10) and ‘high’ (N_KKU_ = 8, N_Huh_ = 7, N_Hep_ = 7) EAI networks. (**B**) Invasiveness in collagen networks with fibronectin addition of different liver cancer cells in ‘low’ (N_KKU_ = 4, N_Huh_ = 5, N_Hep_ = 5), ‘intermediate’ (N_KKU_ = 6, N_Huh_ = 6, N_Hep_ = 7) and ‘high’ (N_KKU_ = 4, N_Huh_ = 6, N_Hep_ = 5) EAI networks. (**C**) Liver cancer cell invasion depth for invasion into pure collagen networks. (**D**) Invasion depth of liver cancer cells in collagen networks with fibronectin addition. Data of the biological replicates N presented as combined box and strip plots. (**E-G**) Cell line-specific invasiveness for (**E**) KKU-213, (**F**) HepG2, and (**G**) Huh7 cells in collagen and collagen-fibronectin networks. (**H-J**) Cell line-specific invasion depths for (**H**) KKU-213, (**I**) HepG2, and (**J**) Huh7 cells in collagen and collagen-fibronectin networks. Boxes are confined by 25th and 75th percentile, horizontal lines are the medians, whiskers describe 5th and 95th percentile. The mean value is indicated by a + symbol. Significance notions were derived from Welch’s unequal variance t-test, ***p ≤ 0.001, **p ≤ 0.01, *p ≤ 0.05. One-way ANOVA test revealed *** significance for each condition shown in (**A-D**). Number of independent repetitions (biological replicates) are indicated as N_KKU_ (KKU-213 cells), N_Huh_ (Huh7 cells), N_Hep_ (HepG2 cells)

KKU-213, Huh7 and HepG2 cells in ‘low’ ECM model systems are predominantly weak invasive in pure collagen and collagen-fibronectin networks exhibiting low invasiveness and invasion depth (Fig. 3A–D). Compared to ‘low’ networks, ‘intermediate’ networks significantly promote the invasion of KKU-213 cells independent of fibronectin addition whereas the invasion of HepG2 cells is not affected by ‘intermediate’ collagen networks with or without fibronectin. In case of Huh7 cells fibronectin addition in ‘intermediate’ networks significantly promoted the invasion compared to ‘low’ networks pointing towards a certain fibronectin-dependent invasion-promoting effect. Distinct differences between the observed cell lines are measurable for invasion into the ‘intermediate’ ECM model systems with and without fibronectin. In networks with ‘high’ EAI, the invasiveness and invasion depth of all observed cell lines were considerably higher compared to ‘low’ and ‘intermediate’ networks. This emphasizes the influence of structural architecture on invasion behavior and the resulting increased aggressiveness of the cells (Table 2).

Huh7 cells are more invasive in networks with additional fibronectin under ‘intermediate’ and ‘high’ conditions compared to pure collagens. In KKU-213 cells and HepG2 cells, no effect on invasion of additional fibronectin in the collagen networks compared to pure collagen was observed. (Fig. 3E–J).

Overall, the altered structural architecture has a promoting effect on the invasion of the cell lines examined. This effect is particularly pronounced in ‘high’ networks with and without fibronectin.

### Cell-matrix interactions within network structures

**Fig. 4:**
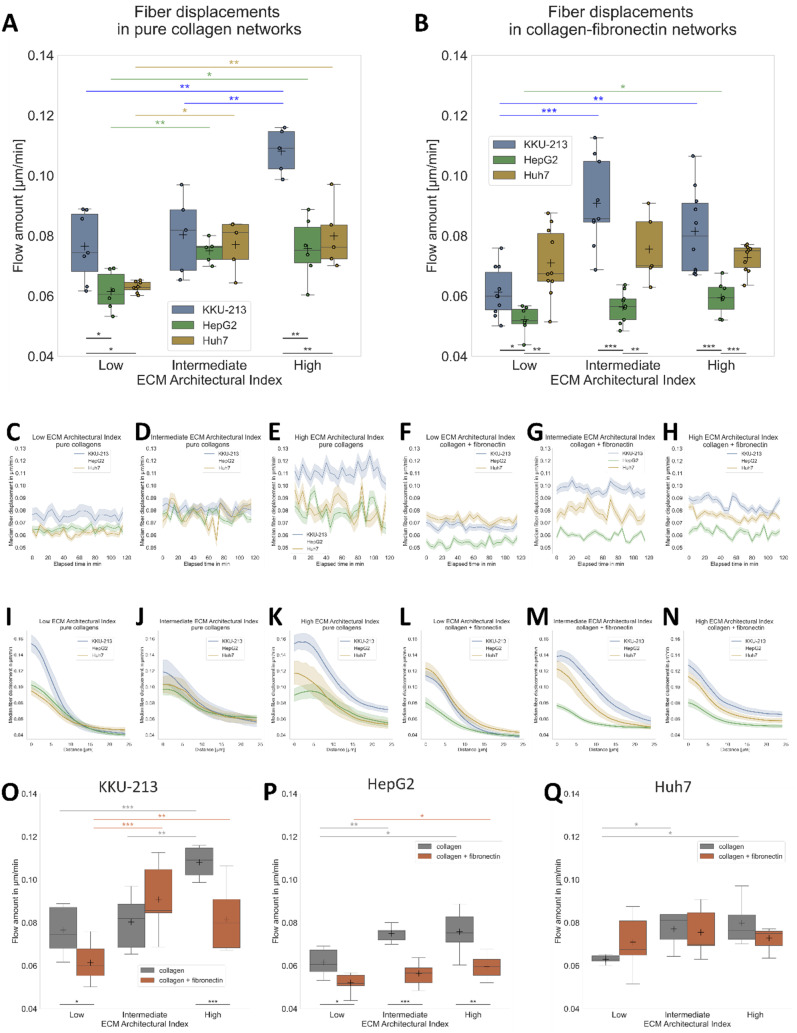
Fiber displacements mirroring cell-matrix interactions (**A**) Fiber displacements of liver cancer cells in pure collagen networks with ‘low’ (n_KKU_ = 37, N_KKU_ = 7, n_Huh_ = 45, N_Huh_ = 8, n_Hep_ = 53, N_Hep_ = 6), ‘intermediate’ (n_KKU_ = 20, N_KKU_ = 5, n_Huh_ = 13, N_Huh_ = 5, n_Hep_ = 37, N_Hep_ = 5) and ‘high’ (n_KKU_ = 20, N_KKU_ = 5, n_Huh_ = 16, N_Huh_ = 5, n_Hep_ = 18, N_Hep_ = 6) EAI. (**B**) Fiber displacement of liver cancer cells in collagen-fibronectin networks with ‘low’ (n_KKU_ = 78, N_KKU_ = 8, n_Huh_ = 86, N_Huh_ = 9, n_Hep_ = 30, N_Hep_ = 7), ‘intermediate’ (n_KKU_ = 56, N_KKU_ = 9, n_Huh_ = 32, N_Huh_ = 5, n_Hep_ = 92, N_Hep_ = 9) and ‘high’ (n_KKU_ = 58, N_KKU_ = 8, n_Huh_ = 70, N_Huh_ = 9, n_Hep_ = 60, N_Hep_ = 7) EAI. Data of the biological replicates N presented as combined box plots and strip plots. (**C-E**) Cell line-specific fiber displacement analyses for (**C**) KKU-213, (**D**) HepG2, and (**E**) Huh7 cells in collagen and collagen-fibronectin networks with varying EAI. Boxes are confined by 25th and 75th percentile, horizontal lines are the medians, whiskers describe 5th and 95th percentile. The mean value is indicated by a + symbol. Significance notions (derived from Mann-Whitney U test) express ***p ≤ 0.001, **p ≤ 0.01, *p ≤ 0.05. Kruskal–Wallis test revealed *** significance for pure collagen as well as for collagen fibronectin networks. Number of replicated measurements (technical replicates) are indicated as n_KKU_ (KKU-213 cells), n_Huh_ (Huh7 cells), n_Hep_ (HepG2 cells). Number of independent repetitions (biological replicates) are indicated as N_KKU_ (KKU-213 cells), N_Huh_ (Huh7 cells), N_Hep_ (HepG2 cells). (**F-K**) The dynamics of the displacements are represented by the median fiber displacements over the elapsed time for (**F**) ‘low’, (**H**) ‘intermediate’ and (**J**) ‘high’ EAI networks for pure collagen ECM model systems and (**G**) ‘low’, (**I**) ‘intermediate’ and (**K**) ‘high’ EAI networks for collagen-fibronectin ECM model systems. (**L-Q**) Median fiber displacements in dependency of the distance from the observed cell for (**L**) ‘low’, (**N**) ‘intermediate’ and (**P**) ‘high’ EAI networks for pure collagen ECM model systems and (**M**) ‘low’, (**O**) ‘intermediate’ and (**Q**) ‘high’ EAI networks for collagen-fibronectin ECM model systems

Observations of fiber displacements in ‘low’ networks consisting of pure collagen revealed differences between the relatively higher displacements for KKU-213 cells and the less pronounced displacements for HepG2 and Huh7 cells. In ‘intermediate’ ECM model systems made of pure collagen HepG2 and Huh7 cells displaced the fibers higher compared to ‘low’ networks whereas the displacements of KKU-213 cells remained in the same displacement-ranges with a small tendency to higher displacements. In pure collagen ECM model systems with ‘high’ EAI, the displacements measured for KKU-213 cells were significantly higher whereas the displacements of HepG2 cells and Huh7 cells remained at the level of the displacements measured in ‘intermediate’ collagen networks (Fig. 4A).

In ‘low’ collagen-fibronectin networks Huh7 cells displaced the fibers with the relatively highest, KKU-213 cells with lower and HepG2 cells with the lowest displacements. In ‘intermediate’ collagen-fibronectin networks the displacements of all investigated cell lines were higher compared to the ‘low’ collagen-fibronectin networks. In collagen-fibronectin networks with ‘high’ EAI the displacements of KKU-213 cells were relatively highest compared to Huh7 cells and to HepG2 cells that displaced the fibers with the relatively lowest displacements (Fig. 4B).

The fiber displacements of the cell lines examined in the respective ECM model systems are independent of each other in terms of time and cell-distance (Fig. 4C–N).

For KKU-213 cells fibronectin-dependent significant differences for ‘low’ and ‘high’ ECM networks were measured. In both cases KKU-213 cells displaced the fibers higher in pure collagen networks (Fig. 4O). Thus, KKU-213 fiber displacements were restricted due to fibronectin within the networks. For HepG2 cells, the presence of fibronectin in the network structures significantly restricted fiber displacement in all ECM model systems (Fig. 4P). For Huh7 cells the displacements in ‘low’ ECM model systems differed significantly between collagen and collagen-fibronectin networks. Fibers in ‘intermediate’ and ‘high’ ECM model systems were displaced at the same levels for pure collagen and collagen-fibronectin networks (Fig. 4Q). Thus, for Huh7 cells the influence of fibronectin to the fiber displacements in ‘intermediate’ and ‘high’ ECM model systems was negligible (Table 2).

**Table 2:** Cell stiffness, cell invasion and fiber displacement measurements. Values describing the boxes (Figs. 2, 3 and 4) in form of 25 percentile (25%), median (50%) and the 75^th^ percentile (75%). The number of independent measurements is N and n is the number of the experimental procedures

EAI	ECM-protein	Cells	Young’s Modulus [Pa]					Invasive cells [%]			Invasion depth [µm]			Invasion		Flow amount [µm/min]				
			25%	50%	75%	N	n	25%	50%	75%	25%	50%	75%	N	n	25%	50%	75%	N	n
Low	Collagen	HepG2	204	232	299	7	21	21	22	24	30	34	44	5	13	0,057	0,061	0,067	6	53
		Huh7	404	479	572	8	24	22	27	28	36	60	64	5	7	0,062	0,063	0,065	8	45
		KKU-213	584	740	908	4	13	24	24	25	36	40	40	5	8	0,067	0,073	0,085	7	37
	Collagen + fibronectin	HepG2	232	290	370	4	19	23	24	26	34	36	40	5	10	0,051	0,052	0,056	7	30
		Huh7	415	472	526	4	11	24	25	26	40	48	48	5	9	0,065	0,067	0,081	9	86
		KKU-213	524	607	754	4	15	25	27	29	38	41	48	4	8	0,055	0,060	0,068	8	78
Intermediate	Collagen	HepG2	252	304	341	6	22	20	23	27	29	34	44	10	28	0,072	0,076	0,076	5	37
		Huh7	194	308	398	5	13	25	30	37	30	56	68	9	21	0,072	0,081	0,084	5	13
		KKU-213	157	178	257	4	13	46	55	56	81	108	114	8	22	0,069	0,082	0,089	5	20
	Collagen + fibronectin	HepG2	327	373	409	4	21	23	25	31	34	44	49	7	14	0,052	0,057	0,059	9	92
		Huh7	387	435	683	5	15	36	40	42	62	78	114	6	12	0,069	0,070	0,085	5	32
		KKU-213	242	422	473	5	15	47	51	53	74	91	108	6	12	0,085	0,086	0,105	9	56
High	Collagen	HepG2	248	313	325	5	20	42	49	53	58	84	108	7	20	0,071	0,075	0,083	6	18
		Huh7	209	220	237	4	15	41	45	50	75	94	102	7	17	0,072	0,076	0,084	5	16
		KKU-213	192	259	263	5	15	58	60	65	120	132	147	8	19	0,102	0,109	0,115	5	20
	Collagen + fibronectin	HepG2	163	190	231	5	24	42	50	56	68	80	112	5	8	0,056	0,059	0,063	7	60
		Huh7	185	206	219	5	19	48	52	53	95	115	128	6	9	0,069	0,075	0,076	9	70
		KKU-213	181	222	300	5	13	49	56	65	85	102	127	4	6	0,068	0,080	0,091	8	58

## Discussion

Alterations in the matrisome play a pivotal role in both tumorigenesis and tumor invasion. Matrisome alterations are driven by multiple interrelated mechanisms, including immune responses, genetic mutations, and structural and compositional changes in the ECM through matrisome dysregulation. Collagen is the structural backbone of the matrisome, providing tensile strength, stability, and a scaffold that organizes and supports other extracellular matrix components. We established tunable collagen-based ECM model systems with different structural architecture that simulate liver tissues with different extend of extracellular remodeling. We have identified a direct correlation between the structural architecture of the ECM model system, cell stiffness, and the aggressive invasive behavior of liver cancer cells. The comparison of purely collagen-based and collagen-fibronectin-based ECM model systems enabled the detection of cell line-specific dependencies in the investigated cell-matrix interaction spectrum.

### Structure and applicability of the ECM model systems

In our study we used different collagen type-I based networks with and without fibronectin to mimic the ECM in different states during liver disease progression. Described for fibrosis and cirrhosis, as driving factors for hepatic disease progression, the ECM changes due to remodeling processes. Thus, the ECM is affected in terms of altered stiffness, pore size, distribution and composition of the network components [34–36]. The stiffness of the ECM influences cancer progression, the mechanotransduction of the cells and thus has an enormous incidence to cell-matrix interactions [36–38]. Notably, ECM composition plays a dual role: highly disorganized matrices rich in collagen create physical barriers that trap natural killer cells and suppress immune surveillance [1, 2], while homogeneous ECM architectures reduce cancer stem cell enrichment and drug resistance [3, 11]. These findings suggest targeting liver ECM structure could synergize with immunotherapies by normalizing matrix stiffness and disrupting immunosuppressive niches [1, 2, 4]. The ECM model systems we use attempt to mimic changes in extracellular conditions by incorporating stiffness and intrinsic factors to describe network properties. These properties were consolidated into a single index (EAI), which quantifies the differences using a score (see Fig. 1). The measured properties of the networks, particularly stiffness, do not increase linearly with rising EAI. Rather, overall structural properties are decisive. In particular, the distribution of network components plays a role, as this was considered within a range (30 µm in every spatial direction, based on an estimated cancer cell diameter of 10–30 µm [39]) relevant to cells, i.e., their immediate surroundings. The different properties of the structures within the networks, particularly the large, node-like structures of the EAI- ‘high’ networks, are attributable to the polymerization of the differently extracted collagens. Bovine collagen obtained through pepsin treatment exhibits limitations regarding its telopeptide sites [40, 41]. This influences the assembly of the collagen components during the reassembly into network structures. The addition of fibronectin prior to collagen polymerization alters the cross-linking behavior of the reconstituting collagen fibers. This leads to fiber alignment and the associated stiffening of the networks, as well as a change in intrinsic properties, as observed elsewhere [42]. In networks with a ‘high’-EAI score and fibronectin, the lower stiffness compared to pure collagen matrices with a ‘high’-EAI score is attributable to the altered structural distribution of the network components, as evidenced by the smaller pore size and broader inhomogeneity distribution. Since stiffness measurements are taken randomly in uniform grids, the measured values reflect the interplay between stiff, node-like regions and softer regions between these node-like structures. In the ‘high’ state, the effect of fibronectin addition is therefore particularly pronounced. In the intermediate network, the effect of fibronectin-induced changes in the network structure is discernible, but it is limited by the more homogeneous distribution of network components compared to the EAI- ‘high’ state. Consequently, the EAI-‘intermediate’ network tends to impart opposite effects under fibronectin addition compared to the EAI-‘high’ networks.

The influence of matrix-embedded fibronectin on tumors is described as highly variable [43]. Homogeneous ‘low’ EAI network systems with and without fibronectin characterized with low stiffness and small pores served as a kind of starting point prior stronger liver ECM remodeling. EAI- ‘intermediate’ networks describe altered liver ECM conditions due to remodeling processes with increased stiffness, larger pores and in case of fibronectin addition altered distribution of network components and thus corresponds to changes caused by fibrogenesis [44]. In agreement with other studies, the addition of fibronectin to pure collagen increased both stiffness, pore size and inhomogeneity with impact to cell-matrix interactions [33, 42]. However, these ‘intermediate’ ECM model systems only describe a specific form of the remodeled ECM in the liver. In addition, EAI- ‘high’ networks with and without fibronectin that possess intermediate stiffness and are formed by large node-like structures mimic strongly altered ECM structures. The node-like structures of EAI- ‘high’ networks and the inhomogeneous distribution of the network components might be associated with cirrhotic liver tissue that contain nodular and dense fibrous structures [45, 46]. The stiffness of the different networks is in a range that is well suited to highlight differences that can also be found in the *locally* characterized in vivo liver tissue stiffness measured by e.g. Zhao et al. or Calò and Romin et al. and are in line with substrate stiffness used for the analyses of fibrotic microniches done by Liu and You et al. [47–49]. The difference to significantly higher in vivo liver stiffness used for diagnosis as for example, by examinations with elastography, which are determined as bulk stiffness of the entire organ, is methodologically conditioned and is therefore not in conflict with the stiffness used here at the cellular level [50, 51].

### Structural influences on cell stiffness and invasion

Different cells or cell lines possess different cellular stiffness in dependence on the substrate they adhere to, as cellular stiffness is regulated by the environment [52, 53]. AFM-based stiffness measurements result in cell-specific stiffnesses. In this way, normal and cancer cells can be distinguished from each other or cancer cell lines with different degrees of aggressiveness [54, 55]. The indentation of single cells on softer substrates (even at low forces) can be influenced by pressing the cells into the substrate [56]. To minimize such effects, we measured cells attached in larger clusters to the respective fibrillary networks (Fig. 2F) and compared the resulting cell line-specific stiffness associated with the respective networks. The cellular properties resulting from the interaction of the ECM model systems are influenced by the respective biophysical properties and structure and thus, have an impact on the invasiveness of the cells and their cell stiffness [57]. In particular, the examined KKU-213 and Huh7 cells exhibit a cell stiffness that develops in opposition to the substrate stiffness. Both cell lines exhibit high cell stiffness when in contact with the less stiff matrix with low EAI and lower cell stiffness when in contact with the stiffer collagen networks with intermediate and high EAI. The oppositely expressed cell stiffness of the HepG2 cells suggest different cell-specific mechanisms that influence stiffness. This entirely different pattern of cell stiffness, in which cell stiffness increases with increasing substrate stiffness, is consistent with many existing studies and corresponds to the expected trend when stiffness is considered as a single influencing parameter. The other two cell lines, KKU-213 and Huh7 cells, also do not consistently deviate from this expected trend. When examining cell stiffness in both intermediate states (collagen ± fibronectin), in which substrate stiffness differs significantly, cell stiffness is higher upon contact with stiffer networks (with fibronectin) than upon contact with softer substrates (without fibronectin). This finding reaffirms the importance of considering the architectural properties (stiffness plus intrinsic properties) of ECM model systems when investigating cell-matrix interactions.

The influence of reduced cell stiffness on invasion into different networks is cell line specific. The structural changes in the networks have a direct effect on the KKU-213 cells. For this cell line, reduced cell stiffness is associated with high invasion in all conditions. This correlation is less pronounced for Huh7 cells. HepG2 cells are less sensitive to cell stiffness changes in contact with the different network conditions. Remarkably, HepG2 cells have low cell stiffness compared to KKU-213 and Huh7 cells even in contact with EAI- ‘low’ networks. Low cell stiffness of all cell lines examined in contact with EAI- ‘high’ networks is noticeable. Under these conditions, all cell lines studied are highly invasive. In contrast to the network structure, the origin of the collagen does not appear to have an effect that increases invasiveness (see supplementary Fig. 2). This suggests that the significantly altered interaction of structural properties (as indicated by a high EAI) leads to a decrease in cell stiffness and thus to the expression of cell stiffness at an “invasion-promoting” level. High EAI and migration-promoting cell stiffness led to highly invasive inter-cooperative behavior. Even though single-cell migration was investigated in this study, parallels can be drawn to the behavior of cancer cells within tumors. In their study on breast and cervical tumors, Fuhs and colleagues showed that softer cells initiate cellular streaming and that the softer cells are more motile than more rigid cells within the same tumor [58].

The invasive behavior of tumors is a reflection of their malignancy and aggressiveness [59–61]. Accordingly, the invasiveness of cancer cells is also a measure of their aggressiveness. The increased invasiveness of all investigated cancer cell lines in EAI-‘high’ ECM model systems is associated with the combination of cellular properties and the properties of the networks. All observed cell lines possess cell stiffness in contact with the EAI-‘high’ networks, which is in a regime that promotes cell-matrix interaction due to the increased flexibility of the cells. Structurally, the EAI-‘high’ networks with and without fibronectin are interspersed with node-like structures in which aligned collagen structures are formed. These structures enable the cells to migrate further and in greater numbers than in the cases with the less aligned network structures of the other ECM model systems. This effect of preferential migration of cancer cells along aligned ECM fibers has already been observed in other studies. Fiber alignment is also found in the vicinity of invasive tumors in vivo and cancer organoids in vitro and indicate ECM remodeling-induced aggressive tumor behavior [62–64].

### Cell line-specific influences of network architecture

Some cancer cell lines apparently have a higher potential for aggressiveness than others. For such highly aggressive cancer cells, even minor changes in the ECM structure are sufficient to unleash this potential in the form of increased migration and invasion. Cell stiffness in combination with structural influences of the immediate environment can affect the ability of cells to invade the respective networks. The fact that stiffness has an influence on cell properties such as migration and invasion is consistent with the findings from the studies by Zhang and Zhao et al. in which this was shown for HCC cells [65]. Recent studies using biomimetic 3D hydrogel models reveal that cirrhotic-level ECM stiffness enhances HCC cell proliferation, epithelial-mesenchymal transition, and chemoresistance compared to fibrotic environments [66]. This stiffness-dependent aggressiveness correlates with upregulated pro-metastatic pathways including Wnt/β-catenin signaling and metabolic reprogramming. Single-cell transcriptomic analyses further identify ECM-driven heterogeneity in malignant subpopulations, with laminin and VEGF signaling pathways facilitating vascular invasion through tumor microenvironment remodeling. Emerging evidence demonstrates that ECM stiffness directly potentiates tumor progression through mechanotransduction pathways such as YAP/TAZ activation, while simultaneously reshaping cell-matrix interactions to promote invasive phenotypes [67, 68].

However, disease-related changes in the ECM are not limited to stiffness alone. The increase in heterogeneity is multi-layered. This issue becomes particularly relevant when considering the highly aggressive iCCA cells (KKU-213), which react more aggressive to individual changes in the properties of the networks than the other cells studied. In contrast to HepG2 and Huh7 cells, which exhibit high invasiveness in EAI- ‘high’ networks, KKU-213 cells also respond to the structural architecture of EAI- ‘intermediate’ networks with increased aggressiveness. Applied to the disease development of the various primary liver tumors, represented by the different cell lines in this study, this observation is supported by the aggressively invasive growth of an iCCA along the bile ducts or into the vein branches [69]. The HCC cell line Huh7 become more invasive under the influence of stiffening and an increase in pore size plus inhomogeneity, as it is the case for ‘intermediate’ networks with fibronectin. In contrast to KKU-213 and Huh7 cells, HepG2 cells are highly invasive only when in contact with EAI- ‘high’ ECM model systems.

Cells that migrate in networks are in constant contact with the network structures. Via integrin binding cells connect to the ECM [70]. This enables the cells to further migrate due to mechanotransduction towards the fiber network [38]. The resulting cell-matrix interactions based on the mechanotransduction are predominantly pulling or pushing the fibrils [71]. Cells react to the properties of the matrix surrounding them [53]. In dependence of the characteristics of the different ECM model systems and the specific abilities of the cell lines used the observed fiber displacements differ. Since these cell-matrix interactions can only be assigned to the invasive part of the cell population, they shed light on the interaction of a specific subset of cells with their extracellular environment. The fiber displacements therefore do not explain the general invasiveness of the cell lines, as cancer cell invasion is dependent on significantly more systemic factors, such as proteases, chemokines, growth factors and others, in addition to mechanotransduction [72]. Rather, they showed the ability of the cells to interact with their immediate environment depending on the matrix properties by describing the force generation and motility capability depending on the structure of the networks used. In this context, the results of the fiber displacement analysis showed that altered ECM structural architecture can promote cell matrix interactions.

The conversion of mechanical signals into biochemical signals underlies cell-matrix interactions. The communication that occurs between cells and the ECM via adhesive (e.g., integrins) or non-adhesive (e.g., Piezo1) force sensors is key to understanding the malignant behavior of cells under the influence of structurally different ECM conditions [73–75]. The question of how mechanosensors that play a key role in changes to structural architecture differ from those stimulated by changes in matrix stiffness requires further investigation. In this context, the mechanosensitive cell nuclei are also exposed to changes transmitted by mechanotransducing factors [76]. As a consequence, signaling pathways such as RhoA/ROCK and YAP/TAZ play a crucial role in mechanical regulation [77, 78]. Cell stiffness, nuclear deformation, and invasiveness form an important mechanobiological axis. Changes in nuclei shape suggest that external influences are mediated by differences in the structural architecture of the extracellular matrix (see supplementary Fig. 1).

### The influence of matrix-bound fibronectin to cell-matrix interactions

The fibronectin used in this study, which was added to the collagen mixture prior fibrillation of the networks, influenced the structure of the networks in terms of stiffness, pore size and network inhomogeneity. In addition, the matrix-bound fibronectin had several distinct although cell-specific effects. Particularly noteworthy is the invasiveness-increasing effect on the HCC cells Huh7 in networks with increasing EAI in the presence of fibronectin. These effects on invasiveness were not observed for KKU-213 and HepG2 cells. In contrast, these cells showed inhibitory effects on cell-matrix interactions in the form of fiber displacements in the fibronectin-containing networks, KKU-213 cells in the EAI- ‘high’ networks and especially HepG2 cells in all network conditions. Fibronectin altered the structural architecture of the networks and influenced cell-matrix interactions in specific settings. As this study primarily investigated the structural contribution and the resulting influence on cell-matrix interactions and not the purely cell biological influence of fibronectin, this enormously important connection must be addressed in greater depth in further studies. There appears to be a multifaceted trade-off between factors that promote invasion into the matrix architecture. This becomes particularly evident when comparing the ‘intermediate’ and ‘high’ EAI states of collagen-fibronectin networks, in which increased invasiveness is not associated with stiffness as much as with the interplay of stiffness with intrinsic properties in the structural architecture of the networks.

Both during the development of cancer and in the presence of a malignant tumor, the ECM undergoes constant remodeling processes. As a result, the tissue surrounding the tumor becomes more heterogeneous and subsequently acts as a factor that creates an immune and therapeutic barrier, thereby fostering a tumor-promoting environment [12]. Moving in the opposite direction on the knowledge axis, i.e., from ‘high’ to ‘low’ EAI, our in vitro model system demonstrates that a decrease in EAI (network-softening and more homogeneous structural distribution) would have an aggressiveness-reducing effect on the cells. In line with this interpretation are the increasingly discussed approaches of treatments of the altered ECM in combination with specific tumor treatments. Approaches such as the use of anti-fibrotic drugs that target growth factors or signaling pathways activated during fibrosis development [44], matrix softening in combination with blocking of intracellular mechanical signal transduction [79], the reversal of liver fibrosis through mesenchymal stem cell therapy [80] or inhibition of collagen biosynthesis and overexpression of matrix metalloproteinases [81], are promising. The positive effects of targeted treatment of pathologically altered ECM on overall cancer treatment are evident.

In addition to the fibronectin extension presented in our study, laminin, elastin, fibrinogen, and other important ECM proteins can be introduced into the system in controllable concentrations, either individually or in combination, as an extension of the collagen base. Further heterogeneity-related influences on cell-matrix interactions depending on the nature of the matrisome can thus be investigated.

Limitations of our work are the complexity of the matrisome, which cannot be represented. The use of collagen ± fibronectin is insufficient to capture the full complexity of the interactions between matrix components. This ECM model system was developed to demonstrate the specific effects of structural architecture on cell-matrix interactions and, in this context, to identify cell-line-specific effects that promote aggressiveness. To improve insight into the influence of the ECM structure on cell-specific regulatory mechanisms and the associated malignancy of cancer cells, an approach based on a biological fingerprint that specifically addresses this issue must be pursued. Furthermore, our studies did not distinguish between the biophysical influences and the potentially distinct biochemical drivers within the various ECM model systems. Biochemical drivers of cancer cell aggressiveness in different states of biophysically distinct systems must be addressed in subsequent studies. Due to the use of cancer cell lines, our in vitro study cannot describe the complex behavior of in vivo tumors with the ECM. Rather, the results presented indicate a structurally driven increase in malignant influences on cancer cells, as can be assumed based on in vivo ECM remodeling. It is necessary to extend the investigations to identify functional analogies in human liver tissue using large-scale structural analyses of tissue sections and the use of primary tumor cells in order to verify the results even with greater cell-specific heterogeneity.

## Conclusion

Our study demonstrates how changes in ECM structure influence biophysical properties and enhance aggressive liver cancer cell invasion. The various ECM model systems and their respective effects on cancer cells illustrate how significant and progressive changes in network heterogeneity influence cancer cell invasion. Our work synthesizes mechanistic insights into how the structural architecture of the ECM drives liver cancer invasion and cell-matrix interaction, emphasizing the potential use of ECM structural architecture for individual risk prediction of cancer invasion and metastasis, as well as the potential for therapeutic interventions.

## Material and methods

All authors had access to the study data and had reviewed and approved the final manuscript.

### Cell lines and cell culture

iCCA cell line KKU-213 [82] (Japanese Collection of Research Cell Bank), Hepatocellular carcinoma cell lines HepG2 and Huh7 were cultured in DMEM with low glucose (1 g/l), 10% Fetal Bovine Serum and 1% Penecilin/Streptomycin in an incubator at standard cell culture conditions (with 95% humidity, 5% CO_2_ and 37°C). Cells were harvested at 70% − 80% confluence using Trypsin/Ethylenediaminetetraacetic acid solution.

### 3D collagen network preparation and modulation with fibronectin

Three collagen types were used to generate collagen networks with different structural architecture. Networks termed ‘low’ systems were made from human collagen (Advanced Biomatrix VitroCol, Cellsystems, Cat. No: 5007–20 ML, Troisdorf, Germany, naturally secreted from human neo-natal fibroblast cells, enzymatic extraction, cleaved telocollagen peptide, purity > 99%, containing > 97% type I collagen, remaining portion is type III collagen). For network polymerization the stock solution was treated according to the manufactures protocol. To craft matrices with final collagen concentration up to 2.48 g/l a mixture of 8 parts collagen stock solution was diluted with one-part 10x phosphate buffered solution (PBS). Under pH control 0.1 M NaOH was added dropwise until a final pH of 7.2–7.4 was reached. Sterile water was used to adjust to the final volume. Collagens termed ‘high’ in this work were made of pure bovine collagen (Collagen G Type 1, Cat. No: L7213, Merck SA, Darmstadt, Germany, calf skin, pepsinized). Collagens termed ‘intermediate’ in this study were made as a mixture of rat tail collagen (Collagen R Solution, Cat. No: 47,256.01, Serva, Heidelberg, Germany, rat tail, non-pepsinized) and the bovine collagen used for the ‘high’ networks in a mass fraction of 1:2. Preparation of network polymerization of ‘intermediate’ and ‘high’ networks followed a protocol described in [33, 83–85]. Briefly, a 1 M buffer solution containing disodium hydrogen phosphate (Sigma Aldrich, Cat. No: 71,636), sodium dihydrogen phosphate (Sigma Aldrich, Cat. No. 71,507) and ultrapure water were mixed with the collagen stock solution (pure bovine for ‘high’ or bovine and rat mix for ‘intermediate’ networks) to obtain a final pH value of 7.4, ionic strength 0.7 and final phosphate concentration of 200 mM. All components for buffers, pipette tips and collagen stock solutions were pre-cooled. Preparation of all collagen matrices (‘low’ heterogeneous, ‘intermediate’ heterogeneous, ‘high’ heterogeneous) were done at ice to prevent early polymerization. After mixing and adding the buffer-collagen solution to suitable dishes or plates (depending on the subsequent use) the networks polymerized in an incubator at standard cell culture conditions (95% humidity, 5% CO_2_ and 37°C). For measurements of stiffness 1 ml of the buffer-collagen solution were added into a 34 mm petri dish (TPP, Cat. No: 93,040, Trasadingen, Switzerland), for invasion assays 600 µl were added to each well of a 12-well cell culture plate (TPP, Cat. No: 92,012, Trasadingen, Switzerland). For confocal laser scanning microscopy (Leica TCS SP8, Mannheim, Germany) imaging 250 µl (fiber displacement assay) or 500 µl (pore size assay) were added in each well of a 24-well µ-plate (ibidi GmbH, Cat. No: 82,426, Gräfelfing, Germany). Incubation times for polymerization were 2 h for ‘low’ and ‘intermediate’ and 5 h for ‘high’ networks.

For the network modulation with fibronectin 50 µg/ml fibronectin (Human Fibronectin Protein, Cat. No: 1918-FN-02 M, R&D Systems, Minneapolis, USA) were added to the buffer-collagen solutions prior to polymerization (According to the manufacturer, the purity of the product is >90%, by SDS-PAGE under reducing conditions and visualized by silver stain). Incubation and polymerization times were the same as described above.

After polymerization all collagen networks were rinsed three times with PBS, kept hydrated and stored in an incubator. Prior to the use of the collagen networks in combination with cells the matrices were incubated with DMEM overnight in an incubator. The structural architecture of the networks reconstituted by polymerization varies significantly and depends on the method used to extract the collagens. Collagens extracted enzymatically by pepsinization exhibit telopeptide-restricted structures, as discussed in detail in numerous studies [40, 41]. The absence of telopeptide ends results in short fibrillar structures that behave differently during polymerization than telopeptide-intact collagens.

### Mechanical characterization of ECM model systems

For mechanical characterization of the collagen-based ECM model systems we used an AFM method as described [33, 84, 86, 87]. For measuring the collagen hydrogels with or without additional fibronectin a polystyrene bead (diameter 45 µm, Polybead, Polysciences, Cat. No: 07314, Warrington, PA 18,976, USA) was glued at the top of a tip-less cantilever. The maximum indentation force was 5 nN [84, 86]. The indentation rate was limited to 3 µm/s. At each collagen condition we measured 3–4 random areas (30 µm ×30 µm) including 3 × 3 points per area. For analyzing the force-distance curves we fitted the standard Hertz model to the retract part of the curve [33, 54, 84, 87]. Measurements were conducted in PBS.

### Structural characterization of hydrogels – pore size and inhomogeneity parameter

The pore size analysis was done as described [33, 54, 84]. The pore size, as the median pore-diameter of the collagen-based network structures was determined by using a custom-built python program published by Fischer and colleagues [88]. In more detail, the collagen networks were fluorescently stained with 20 μg/ml 5 (6)-Carboxytetramethylrhodamine N-succinimidylester trade name TAMRA-SE (Sigma Aldrich, Cat. No: 21,955) over night in an incubator. Afterwards, the collagen gels were rinsed three times with PBS. 3D image stacks of 100 µm edge length were recorded with a confocal laser scanning microscope (Leica TCS SP8, Mannheim, Germany) under usage of a 63x water immersion objective. At each sample 5–6 different image stacks were recorded in at least 3 independent measurements.

To determine the inhomogeneity parameter, as introduced by Hayn and colleagues [84], we divided the large (100 µm edge length) image stacks into smaller parts (30 µm edge length). The large, whole image cube as well as each of the smaller parts were analyzed separately to determine the respective number of pores, the collagen volume and the pore size using the same python program [88] as described for the pore size analysis. The relation, as a percentage, for the parameters of the smaller parts and whole image cube were calculated. The Euclidean norm of the standard deviation of each parameter then yields the inhomogeneity [33, 84].

### ECM Architectural Index (EAI)

In order to better classify the joint influence of stiffness, pore size and inhomogeneity of the different networks on the overall structural architecture, the n-fold changes of all three properties were combined in the Euclidean norm.

$$\begin{aligned}&EAI - score \cr & = \sqrt {{{\left( {n - fold\; stiffness} \right)}^2} + {{\left( {n - fold\;pore\;size} \right)}^2} + {{\left( {n - fold\; inhomogeneity} \right)}^2}}\end{aligned} $$

The resulting parameter thus enables a combined representation and visualization of the respective changes in the networks.

### Cell stiffness measurements in contact with hydrogels

To characterize the stiffness of cells in contact or, more precisely, cultured on the network structures served to determine differences of cellular stiffness due to interaction with the respective network. In preparation of these measurements, collagen-based networks with and without fibronectin were crafted as described in the section 3D collagen network preparation and modulation with fibronectin. At the collagens 250.000 cells were seeded and incubated overnight in an incubator at standard cell culture conditions. Prior to the measurements the cells were rinsed at least three times with fresh cell culture medium to remove non-adherent or dead cells and incubated again for at least one hour in an incubator at standard cell culture conditions to relax again after the washing procedure. Cells in contact with the networks were measured with an AFM (Bruker/JPK CellHesion 200, Billerica, MA 01821, USA) and a measuring protocol as described [54, 83]. In detail, a cantilever is modified with a small polystyrene bead (diameter 6 µm, Polybead, Polysciences, Cat. NO: 07312, Warrington, PA 18,976, USA) using M-Bond 610–1 glue and an over-night hardening at 80 °C in an incubator. The bead-modified cantilever was brought into contact with the respective cells on top of the collagen-based networks. Indentations (5 × 5) at 3–4 different areas (30 µm ×30 µm) with a maximum force of 0.5 nN [54, 83] and an indentation speed of 3 µm/s were done at the surface of the cells. Using the Bruker/JPK software, the standard Hertz Model was fitted to the extend part of the force-distance curves. Poisson ratio was set to 0.5 by default, assuming “cells as incompressible material” [55].

### Invasion assays

To investigate cancer cell invasion into ECM model system networks we prepared the respective collagens with or without fibronectin for invasion assays as described in the section “3D collagen network preparation and modulation with fibronectin”. After final polymerization the networks were rinsed three times with PBS and incubated with DMEM overnight in an incubator at standard cell culture conditions. Afterwards, 20.000 cells were seeded at the surface of each network after treatment with 0.125% Trypsin/EDTA solution and incubated for 72 h in an incubator at standard cell culture conditions following a well-established method [33, 54, 83–85]. Cancer cell invasion was stopped by adding a 2.5% glutaraldehyde-PBS cocktail for 20 minutes at 37 °C followed by 3 washing steps with PBS. Afterwards, a solution containing PBS and 4 µl/ml HOECHST to stain the nuclei of the cells was added and incubated at 4 °C overnight. To image the fluorescent stained cell nuclei 10 × 10 image stacks was taken at a fluorescence microscope (DMI6000B, Leica; Wetzlar, Germany) using a 20x objective and an A4 filter cube. In each image stack z-distance of focal planes was 4 µm. Cells below 12 µm were assumed to be invasive. For the analysis, we used a customized Python application based on algorithms for 3D image analysis and filtering.

### Fiber displacement assay and analysis

The method we used for the assay is already published elsewhere [33, 83]. The imaging of liver cancer cells in collagen-based networks that are prepared as described in the section “3D collagen network preparation and modulation with fibronectin” was done using confocal laser scanning microscopy (Leica TCS SP8, Mannheim, Germany) with a mounted incubation chamber. At each well of a 24-well µ-plate (ibidi GmbH, Cat. No: 82,426, Gräfelfing, Germany) containing prepared collagen gels 2000 cells were seeded and incubated overnight in an incubator at standard cell culture conditions. A fluorescent dye (2 µl/ml Vybrant™ DiD, Thermo Fisher Scientific, Cat No: V22887) was added for live cell staining to the culture medium after adherence of the cells. Single cells within the respective networks were imaged every 5 minutes for 2 hours. The 3D image stacks contained the fluorescent signal of the stained cells and the transmitted light.

The analysis of fiber displacements is based on the assumption that the deformation of the scaffold by the migrating cells is related to the forces that the cell exerts on the 3D collagen fiber matrix by pulling or pushing. Thus, the analysis of fiber displacements provides insights into the interaction of cells with their immediate environment and describes the capability of cells to deform the surrounding 3D matrix scaffold. We used a custom-built Python program based on optical flow analysis. The images were separated into cell bodies (fluorescence signal) and surroundings (transmitted light) based on the available channels. Background drift was compensated by subtracting the zero frequency of a Fast Fourier Transform analysis. The median fiber displacements were calculated for each pixel and between each consecutive time frame and analyzed for 25 consecutive areas around the cell, each with an extension of 1 µm. This enables the analysis of fiber displacements over time and depending on the distance from the cell body.

### Statistical analysis

Our measurements were repeated at least three times independently of each other, unless otherwise stated. The number of technical replicates n and biological replicates N is indicated in the respective figure captions and in the tables. Statistical analyses were performed using a one-way ANOVA and Welch’s test for unequal variances. For data that were not normally distributed, the Kruskal-Wallis test and the Mann-Whitney U test were performed. Information on the significance notions and which tests were performed can be found in the respective figure captions.

## Electronic supplementary material

Below is the link to the electronic supplementary material.

Supplementary Material 1

Supplementary Material 2

## Data Availability

All data generated or analyzed during this study are included in this published article. Additional datasets, including raw data, are available from the corresponding author upon reasonable request.
