# Supplementary material for: Structural architecture of collagen and collagen-fibronectin networks is associated with the invasive behavior of liver cancer cells
Source: Cell Oncol (Dordr). 2026 Jun 12;49(3):87. doi: 10.1007/s13402-026-01235-0 (PMC13263367; doi:10.1007/s13402-026-01235-0)
Supplement: Supplementary file 1 — Supplementary Material 1 [file 13402_2026_1235_MOESM1_ESM.docx]

**Supplementary Material**

**Structural architecture of collagen and collagen-fibronectin networks promotes invasive behavior of liver cancer cells**

**Authors:**

Alexander Hayn, Madlen Matz-Soja, Thomas Berg, Florian van Bömmel

Division of Hepatology, Department of Medicine II, Leipzig University Medical Center, Germany

**Nuclear shape analysis**

**Rationale:** We attempted to generate further insights to identify regulatory mechanisms for cell stiffness and invasiveness. The most prominent component in this constellation is the deformability of cell nuclei. Cell stiffness has a mechanoregulatory effect. The background here includes the actin cytoskeleton of cells [1, 2] and mechanosensitive differentiation [3], which in turn influences nuclear stiffness. The deformability of the cell nucleus itself is an important factor in the migration of cells in confined spaces, such as a collagen scaffold. The nucleus, as the largest organelle in the cell, is in itself a limiting factor in cell invasiveness. Thus, cells that are able to influence nuclear deformation have an invasion-advantage [2]. Forces acting on the cell nucleus from outside are mediated by the linker of nucleoskeleton and cytoskeleton complex [4]. There are a huge number of mechanosensitive signaling pathways that are crucial for mechanical regulation. Important examples include RhoA/ROCK and YAP/TAZ [5, 6]. Cell stiffness, nuclear deformation, and invasiveness form an important mechanobiological axis. Signaling pathways, such as YAP/TAZ, play a major role in biomechanical alterations to tumor initiation and/or progression. The analyses, particularly those based on the circularity of the cell nuclei examined, highlight the diversity of cell line-specific changes depending on the extracellular environment in which the cells are located. The effects on cell nucleus of all parameters are relatively similar when comparing conditions with pure collagen and those with collagen + fibronectin, since neighboring EAI networks are present in both cases. However, the overview of changes in circularity across different EAI levels clearly shows that a decrease in cell nucleus circularity median values is observable in networks with elevated EAI. This is an indication of regulatory mechanisms described above, that affect the shape of the cell nucleus and thus expands our understanding of the increased invasiveness of the examined cell lines due to an altered structural architecture in the extracellular environment.


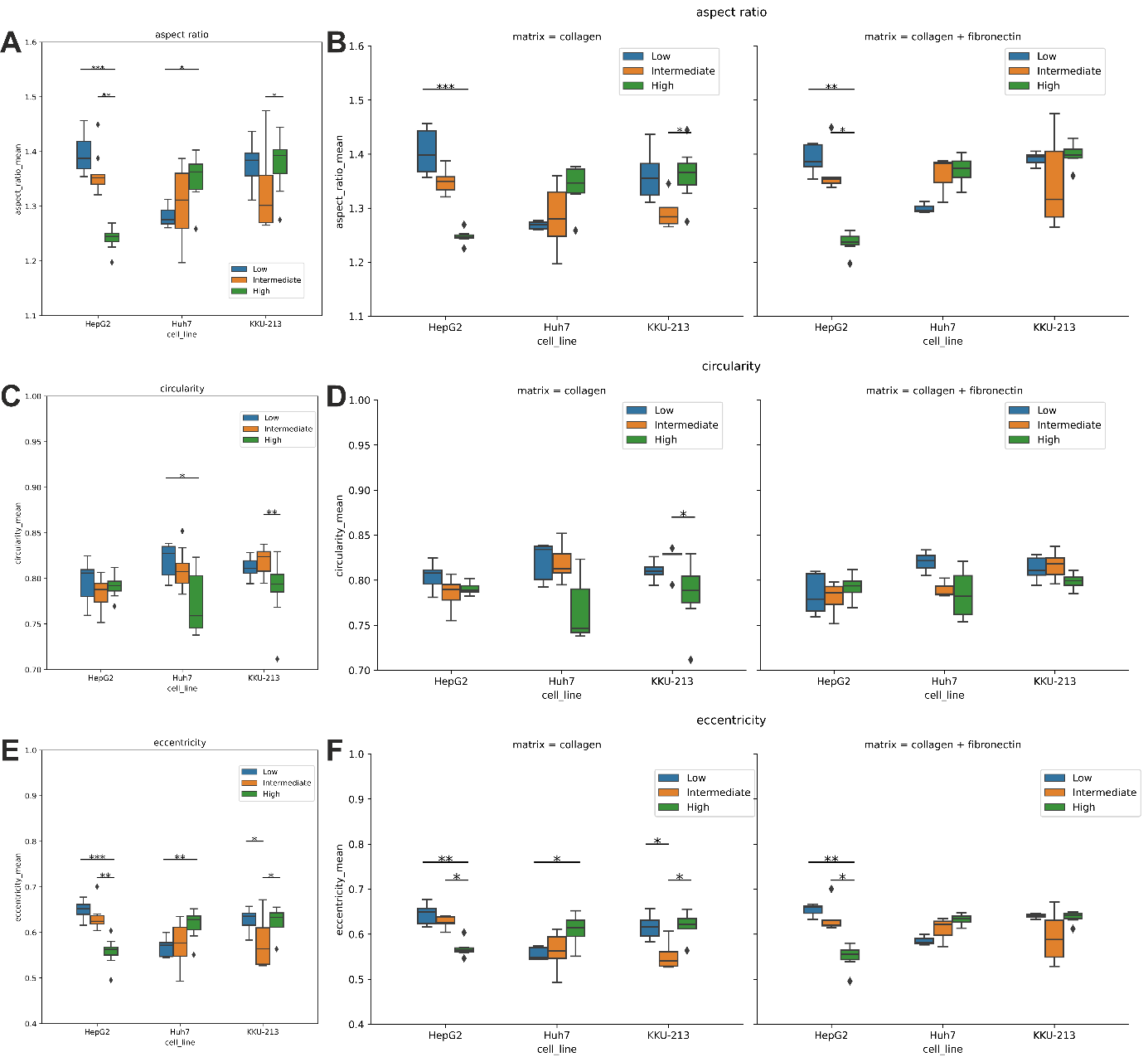


Figure 1 Nuclear shape analysis. **(A)** Aspect ratio of cell nuclei in contact with ECM model systems with different structural architecture. An ECM structural index (EAI) with the categories low, intermediate, and high indicated the different structures and the definitions in the figure legends. **(B)** Differentiation of aspect ratios for ECM model systems based on pure collagen and collagen containing fibronectin. **(C)** Circularity measured for cell nuclei in contact with various ECM model systems. **(D)** Circularity of cell nuclei from different liver cancer cell lines differentiated depending on the presence of fibronectin in collagen-based networks. **(E)** Eccentricity of various liver cancer cell lines in contact with networks of different structures. **(F)** Differences in collagen and fibronectin levels within the networks and in nuclear eccentricity between different cancer cell lines in contact with each of these ECM model systems. Significances derived from non-parametric post-hoc Dunn test after a Kruskal-Wallis-Test.

**Methodology:** Approach to analyzing cell nucleus shape by a custom-built python pipeline: 1. Imaging of HOECHST-stained cell nuclei from at least N=5 conditions, with n=25 images for each cell line and each matrix condition with a Keyence BX-800 fluorescence microscope using a 20x Fluotar objective. 2. Segmentation based on automated selection of the best possible segmentation mask (Otsu, Otsu local, adaptive threshold, Savola) depending on the available fluorescence images. 3. Automated adaptive cluster separation (watershed). 4. Qualitative segmentation check. 5. Exclusion of artifacts and remaining segmentation errors (small spots, remaining clusters, etc.). 6. Exclusion of entire images in the presence of extreme outliers. 7. Cell line-specific deterministic restriction of the analysis to the same number of cell nuclei for each matrix condition. 8. Evaluation of the available images with respect to the median of the parameters (aspect ratio, circularity, eccentricity, etc.) per image 9. Presentation of the averaged parameters of evaluated biological relevant N for each condition.

**Analysis of the influence of network structure versus species-specific differences in extracted collagen on the invasiveness of a liver cancer cell model system**

**Rationale:** The underlying principles governing structural properties and the reason for the differences in network structures do not depend on the animal source of the collagen, but rather on the specific method used to extract the various collagens. The bovine collagen, which produces the highly heterogeneous networks, is obtained from bovine skin by pepsination. We investigated the fundamental question of whether the change in the invasive behavior of the different cells is species-relevant or structure-related by using non-pepsinated bovine collagen to create networks that are structurally different from the pepsinated bovine collagens. After applying consistent collagen concentration (2.5 g/l) and using the established buffer system and consistent polymerization conditions (temperature, pH, etc.), the resulting collagens (named G-telo) were examined for stiffness and intrinsic properties for comparison purposes (see Figure 2). The non-pepsin-treated bovine collagen networks were found to exhibit moderate stiffness, small pore sizes, and a structurally relatively homogeneous distribution of network structures compared to the collagen networks of the ECM model systems. Within the EAI framework, these networks fall roughly at an intermediate level. The invasion of all cell lines in term of invasiveness and invasion depth distribution was less pronounced or similar low to that observed in studies involving mixed collagens with intermediate EAI. A highly invasive pattern of invasiveness and invasion depth, as seen with high-EAI pepsinized bovine collagen, was not observed.

**Methodology:** Collagens termed G-telo were crafted containing non-pepsinized bovine skin collagen (Advanced Biomatrix **TeloCol^®^-10 Type I Collagen #5226,** Carlsbad, CA 92010, USA) similar to the mixed and pure bovine collagens described in the manuscript, using the same polymerization components and conditions. Briefly, a 1 M buffer solution containing disodium hydrogen phosphate (Sigma Aldrich, Cat. No: 71636), sodium dihydrogen phosphate (Sigma Aldrich, Cat. No. 71507) and ultrapure water were mixed with the collagen stock solution (pure bovine for ‘high’ or bovine and rat mix for ‘intermediate’ networks) to obtain a final pH value of 7.4, ionic strength 0.7 and final phosphate concentration of 200 mM. All components for buffers, pipette tips and collagen stock solutions were pre-cooled. Preparation of all collagen matrices were done at ice to prevent early polymerization. After mixing and adding the buffer-collagen solution to suitable dishes or plates the networks polymerized in an incubator at standard cell culture conditions (95% humidity, 5% CO_2_ and 37°C) for 2 hours. Mechanical and structural characterization and cell invasion assays followed the methods described in the manuscript.


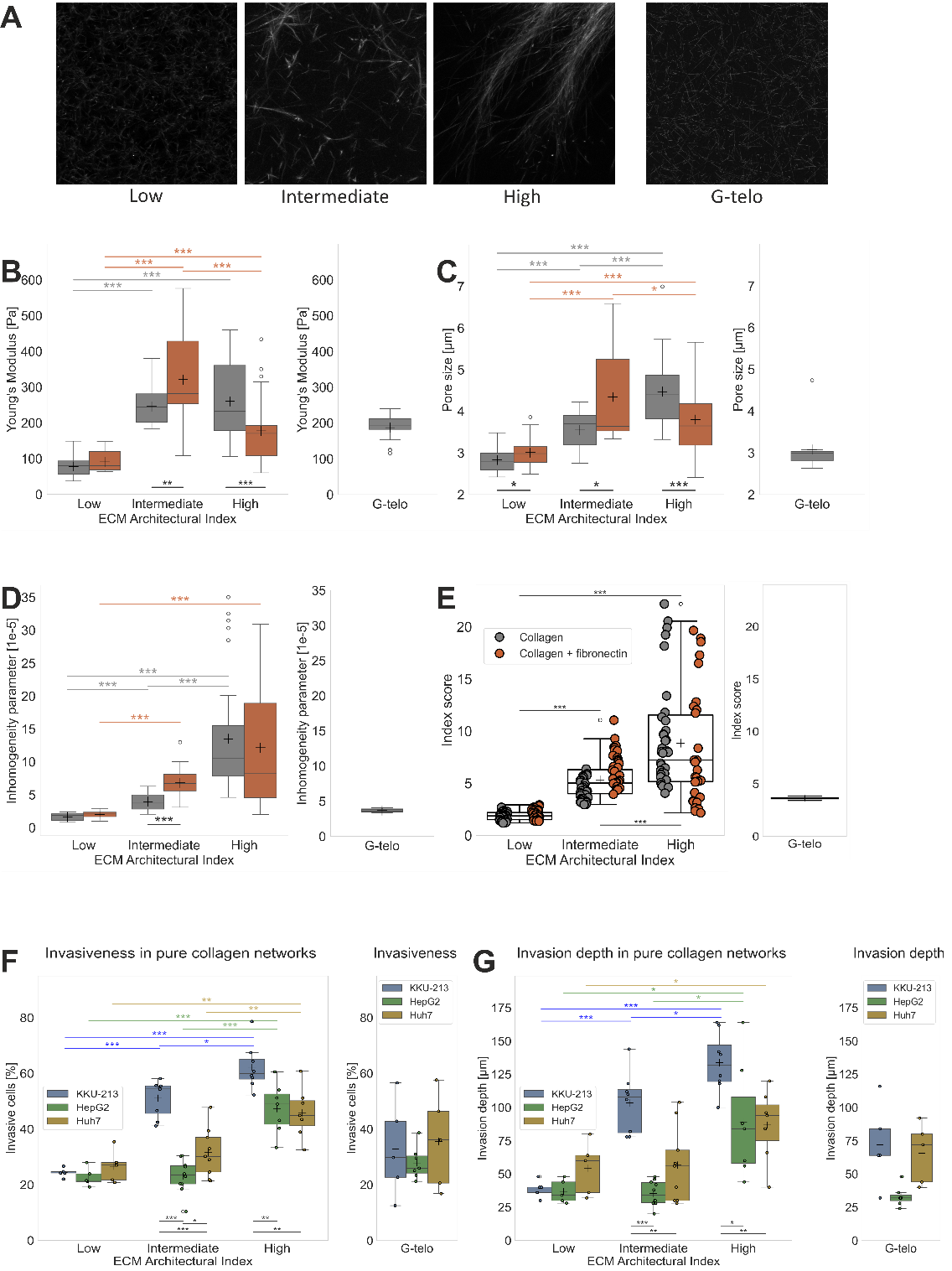


Figure 2 Analysis of the influence of network structure versus species-specific differences in extracted collagen on the invasiveness of a liver cancer cell model system. **(A)** Overview of the network structure based on fluorescence images of stained collagen **(B)** Stiffness measurements **(C)** Pore size analysis **(D)** Distribution of network structures based on an evaluation of inhomogeneity **(E)** Examining the interaction between stiffness and intrinsic parameters on network structure using the ECM Architectural Index **(F)** Invasiveness and **(G)** Invasion depth of different liver cancer cell lines in different collagen-based networks.

References

1. J.-K. Kim, A. Louhghalam, G. Lee, B.W. Schafer, D. Wirtz, D.-H. Kim, Nature communications (2017) doi:10.1038/s41467-017-02217-5

2. T. Fischer, A. Hayn, C.T. Mierke, Frontiers in cell and developmental biology (2020) doi:10.3389/fcell.2020.00393

3. R. Bainer, V. Weaver, Science (New York, N.Y.) (2013) doi:10.1126/science.1243643

4. F. Wei, Y. Liao, A. Wang, X. Zhang, X. Chen, C. Ma, N. Li, J. Chen, M. Long, Med-X (2025) doi:10.1007/s44258-025-00063-5

5. S. Dupont, L. Morsut, M. Aragona, E. Enzo, S. Giulitti, M. Cordenonsi, F. Zanconato, J. Le Digabel, M. Forcato, S. Bicciato, N. Elvassore, S. Piccolo, Nature (2011) doi:10.1038/nature10137

6. J.H. Lim, H.M. Kang, C.-R. Jung, Organoid (2025) doi:10.51335/organoid.2025.5.e4
